# The Relationship with Meeting Physical Activity Guidelines in Preschool-Aged Children: A Systematic Review

**DOI:** 10.3390/pediatric17040079

**Published:** 2025-07-22

**Authors:** Markel Rico-González, Ursula Småland Goth, Ricardo Martín-Moya, Luca Paolo Ardigò

**Affiliations:** 1 Department of Didactics of Musical, Plastic and Corporal Expression, University of the Basque Country, UPV-EHU, 48940 Leioa, Spain; 2 Department of Teacher Education, NLA University College, 0166 Oslo, Norway; ursula.goth@nla.no; 3 Department of Physical Education and Sports, Faculty of Education and Sport Sciences, Campus of Melilla, University of Granada, 52006 Melilla, Spain; rmartinm@ugr.es

**Keywords:** health, childhood, education, sedentary, policymakers

## Abstract

**Background/Objectives**: Physical activity (PA) during preschool is vital for supporting physiological development, enhancing cognitive abilities and fostering socio-emotional growth. However, consistent disparities in meeting PA guidelines have been observed. This systematic review aims to identify studies that compared preschoolers’ PA, as measured by technological devices, with recommended PA guidelines. Specifically, it examines (i) factors associated with meeting PA guidelines and (ii) the outcomes observed when children meet these guidelines. **Methods**: The search strategy was designed based on the PICOS framework. Then, a systematic review was conducted using four databases to identify studies that included children from 0 to 6 years old participating in PA sessions recorded through technological devices. PA is compared with guidelines, and correlations were reported. **Results**: Of the 52 studies reviewed, most found that meeting PA guidelines in preschool-aged children was linked to favourable outcomes across multiple domains. Children who met the guidelines tended to show better motor competence, emotional regulation and cognitive skills, particularly in areas like working memory and social understanding. However, the relationship with body composition and body mass index was inconsistent, suggesting that the benefits of PA in early childhood extend beyond weight-related measures. **Conclusions**: Meeting PA guidelines in early childhood is strongly associated with cognitive development, emotional regulation, motor skills and social behaviours. However, adherence varies significantly due to a complex mix of individual, familial, socioeconomic and environmental factors.

## 1. Introduction

Physical activity (PA) is a fundamental component of early childhood development, influencing various physiological, cognitive and socio-emotional dimensions of health [1]. During the formative years of childhood, habits surrounding PA can lay the foundation for future health behaviours and outcomes. Moreover, PA is of particular interest given the increased prevalence of sedentary lifestyles and related health disorders in children [2].

Given the importance of PA, preschool-aged children, typically defined as children between the ages of 3 and 5, represent a critical demographic, as habits ingrained during this period can determine lifelong behaviour patterns [3]. The concept of the “movement behaviour paradigm” has emerged in recent years. This refers to a holistic framework for understanding how different movement-related behaviours—PA, sedentary behaviour and sleep—interact and collectively influence health and development. This paradigm highlights the importance of taking a holistic approach to fully grasp the collective impact of these behaviours on health and development [4,5]. While initial studies have examined these relationships in older children and adults, comprehensive investigations focusing specifically on preschool-aged children remain limited [4,6,7].

To understand how much PA preschool-aged children perform, the World Health Organisation (WHO) [1] and various national health entities have established guidelines outlining the minimum PA levels recommended for children in this age group. These recommendations have been implemented in countries like the United Kingdom [8], the United States [9], Australia [10], Canada [11] and Spain [12]. Adherence to these guidelines indicates better health outcomes and foundational developmental achievements [13]. However, while the benefits of PA in older children and adults are extensively studied and well-documented, there is a research gap in the understanding of the holistic impact of meeting these guidelines in preschool-aged children [14]. This gap is exacerbated by insufficient data on actual activity levels in this demographic and the diverse factors influencing their compliance with these recommended guidelines [15]. Therefore, it has become crucial to understand the factors affecting adherence to PA guidelines [16].

To foster healthy habits among preschoolers, teachers and professionals working with children under five should be aware that the scientific literature has shown that some factors influence children’s adherence to PA guidelines. Among these factors, gender is a critical yet often overlooked factor [17]. As a social construct, gender intersects with various facets of human behaviour, including health practices [18]. From toy selection to playgroup participation, boys and girls often experience subtle and explicit nudges towards different pathways, which may influence their activity patterns [19]. While some studies [20,21] suggest that boys tend to be more active than girls, engaging in more vigorous or competitive activities, the reasons for these differences and their implications are not fully understood. Are these patterns the result of inherent preferences, societal influences or a combination of both? Furthermore, preschool age is characterised by rapid cognitive development, during which children begin to internalise societal norms and gender roles [22]. The influence of these internalised beliefs on their PA patterns is exciting.

In addition to gender, parental influence, particularly in education, lifestyle, knowledge and attitudes, significantly shapes preschoolers’ activity levels [23,24]. According to Chen et al. [25], higher parental education often correlates with a higher socioeconomic status, providing more opportunities for preschoolers to participate in organised sports, access play areas and enrol in PA programmes. In this context, Rhodes et al. [23] demonstrated that parental attitudes towards PA, influenced by educational experiences, can impact the prioritisation and encouragement of PA among their children. From the preschoolers’ point of view, they often emulate their same-sex parent or the adult in their lives. If the same-sex parent is physically active, the child may be more active [26]. Therefore, all identified correlations should be analysed through a systematic review to encourage preschoolers to adhere to published guidelines (e.g., WHO, Canadian and Australian guidelines).

Until now, these types of information have been analysed using non-objective tools [27,28,29,30,31,32,33]. The scientific literature has established that assessing the amount of PA performed in a valid and reliable way is essential to determine whether or not the daily PA habits align with international guidelines. In this sense, questionnaires have been established as valuable tools [34], since they are easy to carry out and can be used to measure the amount of PA performed in different populations [35]. However, technological devices have introduced the possibility of extracting objective measures, leading to the re-evaluation of traditional assessment methods such as parental reports and observational techniques, which have limitations, such as a lack of precision required for detailed analysis [36,37].

Specifically, some years ago, the emergence of technologies experienced exponential growth in some scientific fields, where educational settings are not an exception [38]. Although Global Navigation Satellite Systems (GNSSs) have been used with preschool children [39], accelerometers are one of the most widely used technological devices [21,38,40]. Accelerometers are wearable devices that measure the intensity and frequency of movement and offer a promising alternative [21] to extract information about PA among preschoolers. Accelerometers provide a more quantifiable and objective perspective, capturing nuances in movement that could be overlooked or misinterpreted in subjective assessments. Their capacity to distinguish between sedentary behaviour, light PA and vigorous PA can provide valuable insights into the quality and quantity of PA in this young age group [40,41].

In light of these considerations, the need to compare children’s PA levels and PA guideline recommendations and identify those factors that lead children to meet suggestions has become crucial. The rationale is mainly to foster a healthy lifestyle from early childhood, a critical period in a person’s lifespan. In this sense, since objective measures have been introduced, there has been an exponential increase in the number of articles that have analysed the amount of preschoolers’ PA levels through accelerometry [38,40]. Other authors, instead, have tried to examine associations between accelerometer-derived PA and some outcomes, such as adiposity-related variables in preschool children [42]. However, regarding guidelines, some authors have been attempting to compare whether children meet a specific PA guideline, such as the WHO, measured through an accelerometer [43]. However, to the best of the authors’ knowledge, no systematic review has tried to identify related factors when meeting (or not) any PA guideline. For this reason, this systematic review aims to identify studies that compared preschoolers’ PA, as measured by technological devices, with recommended PA guidelines. Specifically, it examines (i) factors associated with meeting PA guidelines and (ii) the outcomes observed when children meet these guidelines. By synthesising existing evidence, the review will address current knowledge gaps and contribute to a more comprehensive understanding of how early adherence to PA guidelines influences developmental trajectories in young children. By exploring the intricacies of accelerometer-derived data, we hope to provide significant evidence that reinforces the importance of early PA, informs public health policies and guides parental and educational practices. The authors of the present systematic review expect it to contribute in two ways. First, the authors expected that this work would contribute to highlighting those correlated factors that science has highlighted, drawing a research path toward what has been performed and what researchers can continue investigating, other factors or whether they influence at the same level in preschoolers with different characteristics. Second, the authors expected to highlight the factors related to education in early childhood that professionals should consider to foster PA from the early years. In addition, these correlated factors could be considered by policymakers to promote preschoolers’ adherence to a healthy lifestyle.

## 2. Materials and Methods

### 2.1. Experimental Approach to the Problem

A systematic review was performed using PRISMA (Preferred Reporting Items for Systematic Reviews and Meta-Analyses) guidelines [44] and guidelines for conducting systematic reviews in sports sciences [45]. The systematic review was registered on PROSPERO (PROSPERO 2025 CRD420251052743).

### 2.2. Information Sources

A systematic search of four central databases—PubMed, ProQuest, SCOPUS and FECYT (which includes Web of Science, CCC, CIDW, KJD, MEDLINE, RSCI and SCIELO)—was performed to identify studies published before 22 May 2025.

### 2.3. Search Strategy

The PICO (Patient, Problem or Population–Intervention or Exposure–Comparison, Control or Comparator–Outcome[s]) design was explicitly used to state the question. The search strategy was used in the databases mentioned above. Where possible, the search was limited to scientific studies/journals and language (see exclusion criteria number 6). No lower date limit was applied for publication. The following search terms were used (see Table 1): (preschool OR kindergarten OR “early childhood”) AND (“meet*”) AND (guidelines) AND (“physical activity”) AND (acceleromet* OR pedometer).

### 2.4. Selection Process

To identify information from the studies, one author downloaded the information (title, authors, date and database) and transferred it into an Excel spreadsheet (Microsoft Corporation, Redmond, WA, USA), where duplicates were removed. Two authors screened the remaining studies to select those that met all inclusion criteria (Table 1). In the process, a third author should decide if the two authors disagreed on whether an article should be included or excluded.

Moreover, when relevant studies not previously identified were also screened identically and further studies that complied with the inclusion-exclusion criteria were included and labelled as “included from external sources”.

### 2.5. Data Extraction

The Cochrane Consumers and Communication Review Group’s data extraction template was prepared using an Excel spreadsheet. The spreadsheet assessed inclusion and exclusion requirements for all selected studies. Full-text studies excluded from the analysis were recorded with reasons for exclusion. All records were stored in the spreadsheet. In this process, the authors who decided whether an article should be included or excluded disagreed in two studies. For this reason, the third author agreed that both should be included because they met all the inclusion criteria.

The following information from included articles was extracted: population (e.g., average age, geographic context), PA measured through technological devices, PA duration, technological device’s information, PA guideline used to compare with children’s PA and correlates.

### 2.6. Quality of Studies

On the one hand, the methodological quality was assessed using the methodological index for non-randomised studies (MINORS) [46]. The MINORS scale is a list containing eight essential points and is expanded to 12 points when the studies to be treated are comparative. In this case, it was assessed considering nine items (out of 18 points) due to the non-applicability (NA) of three because these items aim to determine studies that include a control group. Each section’s score can be from 0 to 2, depending on the quality obtained for each point. The MINORS checklist asks for the following information (2 = high quality; 1= medium quality; 0 = low quality).

## 3. Results

### 3.1. Identification and Selection of Studies

**Figure 1:**
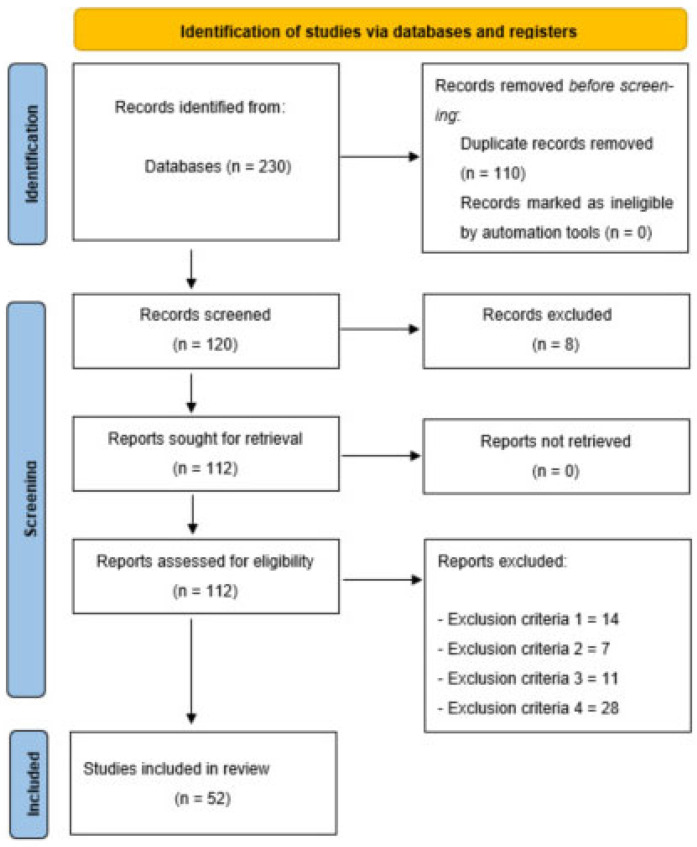
Flow diagram of this study.

Fifty-two studies met the inclusion criteria (Figure 1). Of these, 37 (71%) were cross-sectional and 15 (29%) longitudinal. Sample sizes varied widely: 18 studies enrolled fewer than 200 participants, 24 included 200–999 and 10 involved more than 1000 children. Most participants were from 3 to 5 years old, although some studies also included infants or 6-year-olds. PA was measured objectively in all studies, with 47 (90%) using accelerometers and the remainder employing pedometers or multi-sensor devices. Most studies (*n* = 46) benchmarked activity against the WHO 24 h movement guidelines, while others referred to national standards from Canada, Australia or the UK. Although settings ranged across 28 countries, over half of the studies were conducted in high-income contexts. This heterogeneity in methods and populations enabled broad comparison but posed challenges in synthesis.

### 3.2. Quality Assessment

As assessed using the MINORS checklist, study quality was predominantly moderate to high, with total scores ranging from 12 to 18 out of a possible 18. Of the 52 studies, over 80% scored 14 or above, indicating generally sound methodology (see Supplementary Materials, Table S1 [47,48,49,50,51,52,53,54,55,56,57,58,59,60,61,62,63,64,65,66,67,68,69,70,71,72,73,74,75,76,77,78,79,80,81,82,83,84,85,86,87,88,89,90,91,92,93,94]). However, consistent weaknesses were observed: more than half of the studies did not report a prospective sample size calculation, and nearly all lacked a control group, limiting causal inference. A few studies also scored lower on follow-up adequacy and dropout reporting. These methodological limitations were not evenly distributed; for example, studies with smaller samples and observational designs tended to have lower quality scores. Notably, no clear pattern emerged linking study quality with reported outcomes. However, studies with higher MINORS scores more consistently reported robust associations between meeting PA guidelines and cognitive or motor development. Item 7 (dropout rate) was marked as ‘N/A’ for cross-sectional studies, as they lack a follow-up phase. For longitudinal studies, dropout rates were assessed, with scores reflecting adherence to the <5% threshold (see Supplementary Materials, Table S1).

### 3.3. Information on Included Studies

#### 3.3.1. What Influences Children to Meet PA Guidelines?

Several key factors consistently emerged as correlates of preschoolers’ adherence to PA guidelines:

- Gender: Boys were significantly more active than girls in 38 of the 52 studies (73%), especially in moderate-to-vigorous PA (MVPA).
- Age: 21 studies demonstrated that older preschoolers are more likely to adhere to recommendations.
- Parental influence: factors such as maternal PA levels, education and screen-time regulation were significant pred ictors in at least 19 studies, highlighting the household’s role in shaping movement behaviours.
- Environmental factors: Some environmental factors, such as access to outdoor spaces and rural living, were positively linked to PA adherence in 14 studies.
- Race/ethnicity: Other notable influences included race/ethnicity, with minority children in some settings exhibiting higher activity levels than their majority-group peers, though the reasons for this disparity remain underexplored.
- Socioeconomic status (SES) and parental education showed mixed associations. At the same time, higher SES often correlated with more opportunities for structured PA; some studies noted inverse relationships, possibly due to cultural or contextual factors. Time spent outdoors and reduced sedentary behaviour (e.g., limited screen time) were also strongly associated with guideline adherence (see Supplementary Material, Table S2).

#### 3.3.2. What Are the Consequences of Meeting Physical Activity Guidelines?

Meeting PA guidelines was associated with a range of developmental benefits. Enhanced motor skills were reported in 26 studies, particularly in locomotor and ball-control domains, as measured by tools like the Test of Gross Motor Development-2. Cognitive benefits were evident in 11 studies, with stronger executive function—especially working memory and mental flexibility—among children who met guidelines. Emotional and social advantages, such as fewer behavioural problems and increased prosocial behaviour, were identified in 10 studies, with more pronounced effects in boys (see Table S3)

In contrast, the relationship between PA adherence and body composition was inconsistent. Approximately half of the studies assessing BMI or weight status (13 of 26) found no significant association, suggesting that PA’s benefits in early childhood extend beyond weight-related metrics. For example, while motor and cognitive outcomes showed robust links to PA, BMI results varied by subgroup (e.g., a modest association was found only in preschool girls in one study).

Most studies measured activity over at least five consecutive days using accelerometers, allowing detailed tracking of movement intensity. Contextual factors, such as parental reports of play environments, further enriched the findings. Children meeting PA guidelines demonstrated better outcomes across motor, cognitive and emotional domains, though the strength of these associations varied by context and sex (see Supplementary Materials, Table S3).

## 4. Discussion

This systematic review aims to identify studies that compared preschoolers’ PA, as measured by technological devices, with recommended PA guidelines. Specifically, it examines: (i) factors associated with meeting PA guidelines and (ii) the outcomes observed when children meet these guidelines.

Following this study, the most used PA guidelines are the WHO, Canadian 24 h movement and Australian movement guidelines. A summary of the PA-related recommendations is detailed in Table 2, where similarities can be found, such as at least 30 min of movement in tummy time per day for infants, at least 180 min of PA for toddlers and at least 180 min of PA with one hour of moderate-to-vigorous PA for children older than three years old.

Following the aim of the systematic review, the included studies have been divided into two groups, which are supposed to be the two main sections in the discussion: (1) what factors influence children to meet PA guidelines and (2) what are the consequences of meeting PA guidelines?

### 4.1. What Influences Children to Meet PA Guidelines

Efforts to increase PA among children under five must consider various influencing factors, from individual attributes to structural and contextual determinants. Understanding these can help policymakers, educators and caregivers create evidence-based interventions aligned with WHO recommendations.

#### 4.1.1. Gender and Age

As a social construct, gender intersects with various facets of human behaviour, including health practices. From the selection of toys to participation in playgroups, boys and girls are often nudged towards different pathways subtly and explicitly, which may influence their activity patterns [19]. Numerous studies demonstrated that boys engage in significantly more MVPA than girls [49,50,52,55,56,57,58,63,66,68,70,73,74,78,81,82]. The reasons for these differences and their implications are not fully understood. However, some hypotheses could highlight inherent preferences, societal influences, etc. In addition, since this moment in a person’s life is marked by rapid cognitive development, children begin to internalise societal norms and gender roles [22]. This gender difference is evident across varied cultural contexts, highlighting the persistence of societal and behavioural norms that may favour male participation in active play or structured PA (Leppanen [55], Kim [74] and Vale [80]).

Furthermore, children’s age was consistently identified as another significant predictor of PA levels. In this regard, it was found that older preschoolers are more likely to meet PA guidelines than their younger peers, reflecting developmental progressions in motor skill competence and independence [49,50,52,69,70,78]. For these reasons, it is important to involve all children in attractive actives considering that they could be developmentally appropriate and enjoyable [95].

#### 4.1.2. Race and Ethnicity

Interestingly, race and ethnic background may influence PA engagement, although the direction of this relationship is context dependent. Minority children in some settings exhibited higher levels of light, moderate and vigorous PA than their majority-group counterparts [56,60,70,78]. However, the reasons remain underexplored and may relate to cultural practices, neighbourhood dynamics or targeted interventions.

#### 4.1.3. Parental and Household Influences

SES and household composition further shape children’s PA outcomes. Children from households with low SES, high parity (i.e., later birth order) or single-parent structures tend to have lower PA engagement [56,59,78,96].

Regarding the social determinants of health, the child’s caregivers—especially maternal figures—play an essential role in shaping PA behaviours in two ways: maternal PA level and educational level. On the one hand, multiple studies confirm that maternal activity levels and the strength of the social support network surrounding caregivers directly correlate with children’s activity levels [48,60,69,72,97]. However, when talking about maternal educational level, the findings are not clear. In fact, while in some studies higher maternal education has been associated with increased odds of meeting PA guidelines in some studies [98], other research found an inverse relationship, with children of highly educated parents engaging in less activity (Vale [80]). These contradictory findings point to the complexity of SES as a determinant, with potential moderators such as parental time availability, screen time rules and the value placed on academic vs. physical development.

#### 4.1.4. Environmental and Structural Determinants

Place of residence, including urban vs. rural settings, further modulates PA participation. In South Africa and Tanzania, rural children exhibited higher activity levels than their urban peers, suggesting that access to open play spaces and active transportation may be contributing factors [59,94]. However, this pattern does not hold uniformly, as research in Mongolia found no urban-rural differences.

Structural factors such as access to safe environments, the presence of community recreation areas and early childhood policies also shape PA engagement. Children in resource-poor preschool environments or underfunded education systems tend to be less physically active [59,96], underlining the importance of government-level investments and public policy.

#### 4.1.5. Lifestyle Behaviours and Correlates

Additional behavioural factors influence PA adherence. Time spent outdoors is strongly and positively associated with physical activity, and this relationship appears to be mediated by maternal habits and the time children spend watching television [56,69]. Sedentary behaviour, particularly screen exposure, remains a major barrier to PA participation in preschool-aged children. Conversely, parental life satisfaction or happiness does not show a measurable correlation with children’s PA levels [79].

Moreover, the pattern of PA engagement follows a social gradient—children from more advantaged backgrounds are generally more active [99]. Thus, tackling inequalities in PA requires systemic approaches, such as urban planning to ensure recreational spaces, school policies promoting movement and community-wide initiatives to support families from low-SES backgrounds.

#### 4.1.6. Impact of Sedentary Behaviour on Physical Activity Guideline Adherence

Sedentary time, particularly when structured (e.g., classroom-based activities or screen-based entertainment), may reduce the time available for PA, thus directly affecting adherence to guidelines. In fact, O’Neil et al. [53] found that structured preschool environments often prioritise sedentary activities, such as seated learning, which may inadvertently limit opportunities for movement. This suggests that the context in which sedentary behaviour occurs—at home or in educational settings—plays a critical role in shaping PA patterns.

In this way, several studies included in this review highlight sedentary behaviour, particularly excessive screen time, as a significant barrier to meeting PA guidelines [56,69]. In fact, prolonged screen exposure was consistently associated with reduced MVPA in preschool-aged children, suggesting that sedentary activities may compete with opportunities for active play [56]. This aligns with findings from De Craemer et al. [69], who noted that parental regulation of screen time was a key mediator of PA adherence, with stricter rules linked to higher activity levels. 

Moreover, the relationship between sedentary behaviour and PA is not merely a matter of time displacement. Sedentary behaviours may also influence children’s energy levels, motivation or preference for active play. For instance, excessive sedentary time may contribute to reduced physical literacy or motor skill development, critical for sustained PA engagement [61]. Conversely, interventions that reduce sedentary time, such as incorporating active play zones or movement breaks in preschools, have shown promise in increasing MVPA and overall guideline adherence [86].

The limited focus on sedentary behaviour in the reviewed studies highlights a gap in the literature. Hence, future research should prioritise longitudinal designs to clarify whether reducing sedentary time directly enhances PA levels or if other factors, such as parental modelling or environmental access, mediate this relationship. Such insights could inform targeted interventions, such as preschool policies that balance sedentary learning with structured movement opportunities, to optimise adherence to 24 h movement guidelines.

### 4.2. What Are the Consequences of Meeting Physical Activity Guidelines?

While physical activity is universally recommended for its broad health benefits, its specific impacts on young children remain an area of ongoing research. Findings from the reviewed studies indicate a nuanced picture.

#### 4.2.1. BMI and Physical Health Outcomes

Contrary to common assumptions, the relationship between PA and BMI or weight status in children under five is weak or inconsistent. Most studies found no significant association between PA adherence and body weight or adiposity [54,55,57,59,63,65,66,68,74,75,77,96]. Only one study identified a link between low PA and increased obesity risk in preschool girls [81]. These findings challenge the notion that early childhood PA alone is sufficient to prevent obesity and suggest that BMI is an inadequate marker of child fitness. For example, Guan [54], Lee [77] and Kim [75] found no statistically significant relationship between PA guideline adherence and BMI z-scores. Chaput [68] similarly reported no gender-based adiposity differences despite varying PA levels.

Such discrepancies might be explained by limitations in BMI’s capacity to distinguish fat from lean mass or account for other health dimensions like metabolic fitness. Additionally, dietary patterns, genetic predispositions and environmental exposures (e.g., food insecurity or unsafe play areas) can confound the observed relationships.

#### 4.2.2. Cognitive and Emotional Benefits

More consistent evidence links PA with enhanced emotional and cognitive development in young children. Studies have shown that regular physical activity promotes emotional balance, reduces symptoms of anxiety and depression and supports social-emotional learning [47,58,100]. PA has been positively associated with social cognition, behaviour regulation and general mental well-being in early childhood.

Motor development outcomes—such as motor coordination (MC), locomotor performance and results from assessments like the Test of Gross Motor Development (TGMD-3)—also benefit significantly from higher activity levels [14,49,59,61,62,100]. These findings emphasise that PA is a critical driver of both gross and fine motor skill acquisition, which are foundational for broader developmental competencies.

#### 4.2.3. Language, Learning and Behaviour

Cognitive development in early childhood includes crucial literacy markers like phonological awareness. PA may aid in enhancing phonological working memory and other pre-academic skills [50]. Children meeting PA recommendations also demonstrated improved working memory and lower incidences of externalising behaviours, such as aggression, disobedience and destructiveness [67]. These behavioural outcomes suggest that PA contributes to school readiness and emotional regulation.

#### 4.2.4. Environmental Influences on PA Implementation

Despite its benefits, opportunities for PA within institutional settings, such as preschools and classrooms, may be limited. A study by O’Neil et al. [53] indicates that structured learning environments and classroom factors may reduce children’s physical activity. Sedentary behaviour promoted by prolonged sitting in early education settings could negate some PA gains, reinforcing the need for health-promoting educational practices [101].

In brief, factors such as gender, age, parental influence (maternal PA levels, education and screen-time regulation), environmental factors (access to outdoor spaces and rural living), race/ethnicity and SES could be considered when teachers and professionals working with preschool want to fuel their PA level and subsequently, to achieve cognitive development (e.g., executive function, school readiness), emotional regulation (e.g., emotional well-being), motor skills and social behaviors, which have been correlated when preschool children meet PA guidelines.

However, it is important to note and be critical because all studies differ in the design of their protocols. Among them, studies differ in accelerometer branch (e.g., GT3X+ or ActiGraph GT1M), and since different issues such as sampling frequency [102,103] or the used algorithm [103] can affect it is important to highlight. Another example is the validity and reliability of accelerometers. Even though some of them could be validated [104], it is not easy because different circumstances in different measurement settings could influence their validity (the type of measured PA, the place where the device has been placed, the minimum effort duration and the minimum speed for avoiding unrealistic data) [103]. Another additional difficulty could be the cultural influence on the outcomes of the studies of the different PA guidelines used. These reasons led authors to extend the limitations of the present systematic review.

### 4.3. Limitations

This review presents important insights into the factors influencing PA and its consequences among preschool-aged children. However, several limitations must be acknowledged:

- Most studies included are cross-sectional, limiting the ability to draw causal inferences. While associations between PA and various outcomes are reported, directionality remains unclear.
- More longitudinal, interventional and randomised controlled trials are required to clarify the direction and strength of relationships between PA and child development outcomes. These should utilise objective measurement tools (e.g., accelerometers) and culturally adapted assessment instruments.
- Substantial heterogeneity exists in how studies define and measure PA and guideline adherence. These discrepancies complicate direct comparisons.
- In this way, the methodology of studies that have used technological devices lacks some relevant information, which could be useful due to its impact on the quality of the results obtained during data collection, processing, analysis and reporting [103]. In this way, the authors encouraged the use of previously published surveys [103] to warrant the inclusion of relevant information when using technological devices.
- Numerous studies suffer from limited sample sizes and lack representativeness, often drawing participants from specific schools, geographic locations or socio-demographic strata. This introduces selection bias and undermines the generalizability of findings.
- Studies included in this review span multiple countries and cultural contexts, yet few control for sociocultural variables. Without such contextualization, there is a risk of masking or exaggerating differences between groups.
- Interpreting parental education or SES effects without considering national education systems or social welfare structures may yield misleading conclusions.
- While BMI is commonly used as an outcome, it fails to capture comprehensive aspects of physical health, such as muscle mass, metabolic fitness or physical literacy. Its frequent use may obscure the full benefits of physical activity.
- The systematic review stated that only original studies written in English or Spanish will be included. Future systematic reviews could include original studies written in another language (or without) language restriction.
- Since this systematic review stated that different studies lack of prospective sample size estimation, this calculation is encouraged for future studies.

## 5. Conclusions

The main purpose of this paper was to identify studies that contrasted suggested PA guidelines with preschoolers’ PA as assessed by technological devices. It specifically looked at (i) what influences children to meet PA guidelines and (ii) what outcomes are observed when children meet these guidelines. To maintain the consistency, the conclusion section has been organised in two parts, answering the stated objectives:

- What Influences Children to Meet PA Guidelines?

Adherence varies significantly due to a complex mix of individual, familial, socioeconomic and environmental factors. Boys consistently engage in more PA than girls, and rural or outdoor-rich environments promote higher activity levels than urban settings. Parental influence—particularly maternal modelling—and socioeconomic status also play critical roles, although findings related to education and income are sometimes contradictory, reflecting cultural and contextual variability.

- What Are the Consequences of Meeting Physical Activity Guidelines?

This review demonstrates that meeting WHO PA guidelines in early childhood is strongly associated with cognitive development, emotional regulation, motor skills and social behaviours. While PA is widely promoted for its role in managing childhood obesity, this review finds limited evidence linking PA with BMI or weight outcomes in young children. Instead, the most consistent benefits of PA lie in non-physical domains such as executive function, school readiness and emotional well-being. These findings highlight the importance of broadening the focus of early PA promotion beyond weight management.

## 6. Practical Applications

Guidelines are intended for policy-makers responsible for developing national, sub-regional or municipal plans to increase PA in the population through guidance documents, the education sector, the private sector, research and health care providers.

Key areas for improvement include standardising PA measurement tools, prioritising longitudinal and experimental designs to establish causality and expanding culturally sensitive research in low- and middle-income settings. There is also a need to translate evidence into actionable strategies. Interventions like “Active Play Zones” in early education settings—supported by staff training and daily movement breaks—offer practical, scalable solutions. These designated indoor or outdoor spaces with age-appropriate, low-cost materials (e.g., balls, climbing structures, balance tools) encourage varied movement and social interaction. Combined with staff training and short movement breaks throughout the day, these spaces can significantly increase daily physical activity levels, especially in limited outdoor access.

Policies must also address social inequities by improving access to safe play environments and supporting families from disadvantaged backgrounds.

In sum, ensuring that all preschool children meet PA guidelines is a matter of physical health and a foundation for lifelong learning and development. Future efforts must integrate multisectoral, equity-focused approaches that support active, inclusive environments from the earliest stages of life. In this regard, it is encouraged to be informed about the design approaches, design principles and design recommendations (engaging people of all ages and abilities, selecting play space equipment, accessing play space equipment, grouping play space equipment, incorporating risk and challenge, physical play and sensory play, social play, cognitive/dramatic/imaginative play) for a universal design [105].

## Figures and Tables

**Table 1:** Inclusion and exclusion criteria for study selection.

No.	Item	Inclusion Criteria	Exclusion Criteria	Search Coherence
1	Population	Children from preschool (0–6 years)	Children outside preschool age (e.g., elementary, secondary or higher education)	Preschool OR kindergarten
2	Intervention/Exposure	Physical activity measured using technological devices (e.g., accelerometers, pedometers)	Physical activity not measured or measured without technology (e.g., questionnaires)	Meet*
3	Comparison	Physical activity compared with guidelines	Physical activity not compared with guidelines	Guidelines
4	Outcome(s)	Correlation between meeting guidelines and other factors	No correlation reported between meeting guidelines and other factors	“Physical activity”
5	Study Design	No restrictions	None	–
6	Other Criteria	Peer-reviewed, original, full-text studies in English or Spanish	Non-peer-reviewed, non-original or studies in other languages	–

**Table 2:** Summary of the most considered PA guidelines.

Summary of Guidelines			
24 h GUIDELINES			
	Infants (<1 Year)	Toddler (1–2 Year)	Preschoolers (>3 Years)
WHO	>30 min in tummy time position	>180 min of physical activity	>180 min of physical activity (60 min should be moderate to vigorous)
CANADIAN 24H MOVEMENT	Being physically active in as wide a variety of modes as possible, especially floor games. At least 30 min of tummy time spread throughout in the day while awake for those children wo cannot move.	>180 min of physical activity in as wide a variety of activities of any intensity as possible, which should include energy games, spread throughout the day, the more intense the activity.	>180 min of physical activity in as wide a variety of activities of any intensity as possible, of which at least 60 min should be energy play, the more the better.
**GENERAL GUIDELINES**			
	**Infants (<1 year)**	**Toddler (1–2 year)**	**Preschoolers (>3 years)**
AUSTRALIAN MOVEMENT GUIDELINES	Supervised interactive games on the floor (the more the better). At least 30 min of tummy time throughout the day including movement of arms and legs. Reaching and grasping objects.	At least 3 h per day of varied physical activity including vigorous play.	>3 h per day of varied physical activity including 1 h of vigorous play.

## Supplementary Materials

The following supporting information can be downloaded at: https://www.mdpi.com/article/10.3390/pediatric17040079/s1, Table S1: Methodological Quality Assessment of Included Studies Using the MINORS Checklist; Table S2: Factors Influencing Preschool Children’s Adherence to Physical Activity Guidelines; Table S3: The correlations between meeting physical activity guidelines and their effects.
