# Supplementary material for: The Relationship with Meeting Physical Activity Guidelines in Preschool-Aged Children: A Systematic Review
Source: Pediatr Rep. 2025 Jul 22;17(4):79. doi: 10.3390/pediatric17040079 (PMC12389701; doi:10.3390/pediatric17040079)
Supplement: Supplementary file 1 [file pediatrrep-17-00079-s001.zip › pediatrrep-3714410-supplementary.pdf]

Table S1. Methodological Quality Assessment of Included Studies Using the MINORS Checklist.

Reference	Item 1	Item 2	Item 3	Item 4	Item 5	Item 6	Item 7	Item 8	Item 9	Item 10	Item 11	Item 12	Total Score
Christian et al. [47]	2	2	2	2	2	2	N/A	0	N/A	N/A	N/A	2	14/16
de Lucena Martins et al. [48]	2	2	2	2	2	2	N/A	2	N/A	N/A	N/A	1	15/16
Nicolai Ré et al. [49]	2	2	2	2	2	2	N/A	0	N/A	N/A	N/A	2	14/16
McNeill et al. [50]	2	2	2	2	2	2	0	2	N/A	N/A	N/A	2	18/18
Kracht et al. [51]	2	2	2	2	2	2	0	0	N/A	N/A	N/A	2	14/18
Meredith-Jones et al. [52]	2	2	2	2	2	2	0	0	N/A	N/A	N/A	2	14/18
O'Neill et al. [53]	2	2	2	2	2	2	N/A	0	N/A	N/A	N/A	2	14/16
Guan et al. [54]	2	2	2	2	2	2	0	0	N/A	N/A	N/A	2	14/18
Leppänen et al. [55]	2	2	2	2	2	2	0	0	N/A	N/A	N/A	2	14/18
Chen et al. [56]	2	2	2	2	2	2	0	0	N/A	N/A	N/A	2	14/18
Santos et al. [57]	2	2	2	2	2	2	0	0	N/A	N/A	N/A	2	14/18
Cliff et al. [58]	2	2	2	2	2	2	0	0	N/A	N/A	N/A	2	14/18
Draper et al. [59]	2	2	2	2	2	2	N/A	0	N/A	N/A	N/A	2	16/16
Khalsa et al. [60]	2	2	2	2	2	2	0	0	N/A	N/A	N/A	1	15/18
Hall et al. [61]	2	2	2	2	2	2	0	0	N/A	N/A	N/A	1	13/18
Breau et al. [62]	2	2	2	2	2	2	0	0	N/A	N/A	N/A	1	13/18
Feng et al. [63]	2	2	2	2	2	2	0	2	N/A	N/A	N/A	2	16/18
Kambas et al. [64]	2	2	2	2	2	2	N/A	0	N/A	N/A	N/A	2	14/16
Armstrong et al. [65]	2	2	2	2	2	2	0	0	N/A	N/A	N/A	2	16/18
Berglind et al. [66]	2	2	2	2	2	2	0	0	N/A	N/A	N/A	2	16/18
Carson et al. [67]	2	2	2	2	2	2	0	0	N/A	N/A	N/A	2	16/18
Chaput et al. [68]	2	2	2	2	2	2	0	0	N/A	N/A	N/A	2	16/18
De Craemer et al. [69]	2	2	2	2	2	2	0	0	N/A	N/A	N/A	2	16/18
Slaton et al. [70]	2	2	2	2	2	2	0	0	N/A	N/A	N/A	2	16/18
Lee et al. [71]	2	2	2	2	2	2	0	0	N/A	N/A	N/A	2	16/18
Jago et al. [72]	2	2	2	2	2	2	0	0	N/A	N/A	N/A	1	15/18

Li et al. [73]	2	2	2	2	2	2	0	0	N/A	N/A	N/A	2	16/18
Kim et al. [74]	2	2	2	2	2	2	0	0	N/A	N/A	N/A	2	16/18
Kim et al. [75]	2	2	2	2	2	2	0	0	N/A	N/A	N/A	2	16/18
Larouche et al. [76]	2	2	2	2	2	2	0	0	N/A	N/A	N/A	2	16/18
Lee et al. [77]	2	2	2	2	2	2	0	0	N/A	N/A	N/A	2	16/18
Kracht et al. [78]	2	2	2	2	2	2	0	0	N/A	N/A	N/A	2	16/18
Engberg et al. [79]	2	2	2	2	2	2	0	0	N/A	N/A	N/A	2	16/18
Taylor et al.	2	2	2	2	2	2	0	0	N/A	N/A	N/A	2	16/18
Vale et al. [80]	2	2	2	2	2	2	0	0	N/A	N/A	N/A	2	16/18
Vale et al. [81]	2	2	2	2	2	2	0	0	N/A	N/A	N/A	2	16/18
Vale et al. [82]	2	2	2	2	2	2	N/A	0	N/A	N/A	N/A	2	14/16
Adeta et al. [83]	2	2	2	2	2	2	N/A	0	N/A	N/A	N/A	2	14/16
Mota et al.	2	2	2	2	2	2	0	0	N/A	N/A	N/A	2	14/18
Cristian et al. [84]	2	2	2	2	2	2	0	0	N/A	N/A	N/A	2	14/18
Yin et al. [85]	2	2	2	2	2	2	0	0	N/A	N/A	N/A	2	14/18
Palmer et al. [86]	2	2	2	2	2	0	N/A	0	N/A	N/A	N/A	2	12/16
Sigmundová et al.	2	2	2	2	2	0	0	0	N/A	N/A	N/A	2	12/18
Kolehmainen et al. [87]	2	2	2	2	2	2	0	0	N/A	N/A	N/A	2	14/18
Yu et al. [88]	2	2	2	2	2	2	N/A	0	N/A	N/A	N/A	0	12/16
Ré et al. [89]	2	2	2	2	2	0	N/A	2	N/A	N/A	N/A	2	14/16
Mwase-Vuma et al. [90]	2	2	2	2	2	0	N/A	0	N/A	N/A	N/A	2	12/16
Byambaa et al. [91]	2	2	2	2	2	2	N/A	0	N/A	N/A	N/A	2	14/16
Cristian et al. [92]	2	2	2	2	2	2	0	0	N/A	N/A	N/A	2	14/18
Li et al. [93]	2	2	2	2	2	2	0	2	N/A	N/A	N/A	2	16/18
Nusurupia et al. [94]	2	2	2	2	2	2	N/A	0	N/A	N/A	N/A	2	16/18

Note: MINORS checklist items: 1 = Clearly defined objective, 2 = Inclusion of patients consecutively, 3 = Information collected retrospectively, 4 = Assessments adjusted to objective, 5 = Evaluations carried out in a neutral way, 6 = Follow-up phase consistent with the objective, 7 = Dropout rate during follow-up less than 5%, 8 = Prospective estimation of sample size, 9 = Adequate control group, 10 = Simultaneous groups, 11 = Homogeneous starting groups, 12 = Appropriate statistical analysis. Scoring: 2 = High quality, 1 = Medium quality, 0 = Low quality, N/A = Not applicable.

Table S2. Factors Influencing Preschool Children's Adherence to Physical Activity Guidelines.

Ref.	Sample	What Are Children Doing?	Duration of Data Recording	Technology (Type and Model)	Reference Guideline	How Were the Other Variables Extracted?	Correlation Variables	Results	Conclusions and Practical Applications
de Lucena Martins et al. [48]	Nº children: 270 Schools: 6 preschools Country: Brazil (Mean age 3.97 yrs)	Children activity recorded all day	7 consecutive days, 24h (excl. sleep/water)	Accelerometer (Actigraph wGT3X)	WHO 24-h guidelines	Demographic correlates (including mother's educational level, presence of siblings at home, parents' unemployment and child's primary caregivers) were reported by children's parents/ guardians	Mother's education, siblings, unemployment, caregiver	Child's primary caregiver most associated	Caregiver role critical
Nicolai Ré et al. [49]	Nº children: 1017 Schools: 3 preschools, elementary, middle Country: Brazil (Mean age 7.21)	Children activity recorded all day	7 consecutive days, 24h (excl. sleep/water)	Accelerometer (ActiGraph GT3X+)	WHO 24-h guidelines	Demographic correlates (including age, school level and gender) were collected	Age, school level, gender	Age, school level negatively correlated	Physical activity (PA) declines with age
McNeill et al. [50]	Nº children: 247/185 Schools:	Children activity recorded all day	7 consecutive days, 24h	Accelerometer (ActiGraph GT3X+)	Australian 24-Hour	Demographic correlates (including age, school level and	Age, school level, gender	Girls spent less time in moderate-to-	Gender matters

	preschool Country: Australia (Mean age 4.3)		(excl. water)			gender) were collected		vigorous PA (MVPA)	
Meredith-Jones et al. [52]	Nº children: 547 Schools: N/A Country: New Zealand (Mean age 1, 2 and 5 yrs)	Children activity was recorded during all day	5-7 consecutive days during 24 h	Accelerometer (Actical, Respironics, Murrysville, USA)	Canadian 24-h movement guidelines for the early years (0–4 years)	Demographic correlates (including age and gender) were collected	Age and gender	PA increased with age. At 1, boys performed more PA than girls	Age and gender matter in meeting PA guideline
O'Neill et al. [53]	Nº children: 341 Schools: 3 preschools Country: USA (Mean age 4.6 yrs)	Children activity was recorded during all day	5 consecutive weekdays during 24 h (not considering sleep time or water activities)	Accelerometer (ActiGraph GT1M and GT3X, ActiGraph-Corp, Pensacola, USA)	Early Childhood Obesity Prevention Policies	In-school and out-school time Demographic correlates (including gender) were collected	In-school and out-school time Gender	Children performed more PA out-school Boys performed more PA than girls	In-school PA and gender matter in meeting PA guideline
Leppänen et al. [55]	Nº children: 778 Schools: 66 preschools Country: Finland (Mean age 4.7 yrs)	Children activity was recorded during all day	7 consecutive days during 24 h (not considering water activities)	Accelerometer (ActiGraph wGT3X-BT, ActiGraph-Corp, Pensacola, USA)	Australian 24-Hour Movement Guidelines for the early years (Birth to 5 years)	Demographic correlates (including gender) were collected	Gender	Boys performed more PA than girls	Gender matters in meeting PA guideline
Chen et al. [56]	Nº children: 864 Schools: preschool	Children activity	7 consecutive	Accelerometer (ActiGraph	Canadian and Australian 24-	Demographic correlates (including gender,	Gender, ethnicity, birth	Male sex, Malay ethnicity, higher birth	Maternal behaviours (MBs) as early

	Country: Singapore (Mean age 5.5 yrs)	was recorded during all day	days during 24 h	wGT3X-BT, ActiGraph-Corp, Pensacola, USA)	Hour Movement Guidelines for children and youth	ethnicity, maternal activity and television viewing) were collected	order, maternal activity and television viewing	order and higher maternal activity level were associated with greater moderate-to-vigorous physical activity (MVPA) Maternal activity and television viewing were associated with meeting PA guideline	as during pregnancy could be important targets for future interventions aiming to promote these MBs in children
Santos et al. [57]	Nº children: 202 Schools: preschool Country: Australia (Mean age 1.6 yrs)	Children activity was recorded during all day	7 consecutive days during 24 h	Accelerometer (ActiGraph GT3X, ActiGraph-Corp, Pensacola, USA)	Australian 24-Hour Movement Guidelines for the Early Years (Birth to 5 years)	Demographic correlates (including gender) were collected	Gender	Boys showed less sedentary time than girls	Gender matters in meeting PA guideline
Cliff et al. [58]	Nº children: 248 Schools: preschool Country: Australia (Mean age 4.2 yrs)	Children activity was recorded during all day	7 consecutive days during 24 h	Accelerometer (ActiGraph GT3X, ActiGraph-Corp, Pensacola, USA)	Australian 24-Hour Movement Guidelines for the Early Years (Birth to 5 years)	Demographic correlates (including gender) were collected	Gender	Boys performed more MVPA than girls	Gender matters in meeting PA guideline

Draper et al. [59]	Nº children: 88<br>Schools: 7 preschools<br>Country: South Africa<br>(Mean age 4.5 yrs)	Children activity was recorded during all day	3 consecutive days during 24 h (not considering sleep time or water activities)	Accelerometer (ActiGraph GT3X, ActiGraph-Corp, Pensacola, USA)	WHO guideline for physical activity for children under five years of age	Demographic correlates (including preschool location) were collected	Preschool location	Rural children were more active compared to urban children	Living setting matters in meeting PA guideline
Khalsa et al. [60]	Nº children: 386<br>Schools: 30 preschools<br>Country: USA<br>(Mean age 4.3 yrs)	Children activity was recorded during all day	1 day during 24 h (not considering water activities)	Accelerometer (Actical, Respironics, Murrysville, USA)	Australian 24-Hour Movement Guidelines for the early years (Birth to 5 years)	Demographic correlates (including race) were collected	Race	Black children performed more light, moderate and vigorous physical activity (LMVPA)	Future studies should<br>include an assessment of usual physical activity on weekdays and weekends and should examine the longitudinal effects of adherence to recommendation among preschoolers on body mass index (BMI) and other health outcomes
Feng et al. [63]	Nº children: 251<br>Schools: 8 preschools<br>Country: China	Children activity was recorded	7 consecutive days during 24 h	Accelerometer (activPAL3 micro or ac-	WHO guidelines on physical activity, sedentary behavior, and	Demographic correlates (including gender) were collected	Gender	Boys had higher PA and MVPA than girls	Gender matters in meeting PA guideline

	(Mean age 5.1 yrs)	during all day		tivPAL3ac-tivPAL3C vt, PAL Technologies, Glasgow, UK)	sleep for children less than 5 years of age				
Kambas et al. [64]	Nº children: 250<br>Schools: preschool<br>Country: Greece<br>(Mean age 5.5 yrs)	Children steps were counted during all day	7 consecutive days during 24 h (not considering sleep time or water activities)	Pedometer (Omron Walking style pro HJ-720IT-E2, OMRON, Kjoto, Japan)	Recommended minimum of 10 000 steps/day (Tudor-Locke et al., 2011)	Weekdays and weekend data were collected<br>School-time and leisure-time data were collected<br>Demographic correlates (including body weight and height) were collected	Weekdays and weekend steps<br>School-time and leisure-time steps<br>BMI	Children performed more steps on weekdays than during weekends and during leisure time than school<br>Normal children performed more steps than obese on weekdays, weekend days, during school, after school and weekly	Taking into account the high rate of both the obesity prevalence and children not meeting the 10000 steps/day guideline, the need for preventive policies becomes obvious
Berglind et al. [66]	Nº children: 830<br>Schools: preschool<br>Country: Sweden	Children activity was recorded during all day	7 consecutive days during 24 h (not considering	Accelerometer (ActiGraph GT3X+, ActiGraphCorp,	Canadian 24-Hour Movement Guidelines for Children and Youth	Demographic correlates (including gender) were collected	Gender	Boys accumulated more PA, spending more time in light physical activity and	Gender matters in meeting PA guideline

	(Mean age 4.3 yrs)		sleep time)	Pensacola, USA)				MVPA and less time stationary compared with girls	
Chaput et al. [68]	Nº children: 803<br>Schools: preschool<br>Country: Canada<br>(Mean age 3.5 yrs)	Children activity was recorded during all day	7 consecutive days during 24 h (not considering sleep time)	Accelerometer (Actical, Respironics, Murrysville, USA)	Canadian 24-Hour Movement Guidelines for the Early Years (0-4 years)	Demographic correlates (including gender) were collected	Gender	No significant differences were observed between boys and girls	Gender does not matter in meeting PA guideline
De Craemer et al.[69]	Nº children: 595<br>Schools: 27 preschool<br>Country: Belgium<br>(Mean age 4.2 yrs)	Children activity was recorded during all day	6 consecutive days during 24 h (not considering sleep time)	Accelerometer (GT1M, GT3X and GT3X+, ActiGraph-Corp, Pensacola, USA)	WHO Guidelines on Physical Activity, Sedentary Behaviour and Sleep for Children under 5 Years of Age	Weekdays and weekend data were collected<br>Demographic correlates (including age, parents' screen time, father education and attending a sports club) were collected<br>Body weight and height	Age, weight status, parents' screen time, father socioeconomic status and attending a sports club	Older preschoolers, having a normal weight compared with being underweight, having parents not watching much television and having a father with attained higher education were associated with meeting	Only a few factors were associated with meeting the guideline<br>A more comprehensive measurement of preschool children's potential correlates of PA is warranted.

								guideline on weekdays<br>For weekend days, a significant association was found with attending a sports club	
Slaton et al. [70]	Nº children: 588<br>Schools: 54 preschools<br>Country: USA<br>(Mean age n/a)	Children activity was recorded during all day	2 consecutive days during 24 h	Accelerometer (Actical, Respironics, Murrysville, USA)	WHO Guidelines on Physical Activity, Sedentary Behaviour and Sleep for Children Under 5 Years of Age	Test of Gross Motor Development-2 (TGMD-2)<br>Demographic correlates (including sex, age and race) were collected<br>Body weight and height	Motor competence (MC)<br>Sex, age and race<br>BMIz weight status	The odds of meeting MVPA guidelines were associated with higher MC scores, sex (male), age (older) and race (white), but not with BMIz weight status	Object control competence relates positively to attaining global PA guidelines
Jago et al. [72]	Nº children: 1267<br>Schools: 57 primary schools<br>Country: UK<br>(Mean age 6 yrs)	Children activity was recorded during all day	5 consecutive days during 24 h	Accelerometer (GT3X, ActiGraph-Corp, Pensacola, USA)	UK government guidelines of PA	Accelerometer (GT3X, ActiGraph-Corp, Pensacola, USA)	PA of parents	There were weak associations between the MVPA of 5–6 year old children and their parents	Clinicians and public health professionals should encourage parents to create opportunities for their children to be active

Li et al. [73]	Nº children: 322 Schools: 5 preschools Country:China (Mean age 4.7 [boys] and 4.6 [girls] yrs)	Children activity was recorded during all day	7 consecutive days during 24 h (not considering sleep time or water activities)	Accelerometer (ActiGraph wGT3X-BT, ActiGraph-Corp, Pensacola, USA)	WHO Guidelines on Physical Activity, Sedentary Behaviour and Sleep for Children	Demographic correlates (including gender) were collected	Gender	Boys reported significantly higher compliance with PA recommendations	Gender matters in meeting PA guideline
Kim et al. [74]	Nº children: 421 Schools: 6 preschools Country: Japan (Mean age 4.6 yrs)	Children activity was recorded during all day	7 consecutive days during 24 h (not considering sleep time or water activities)	Accelerometer (Active Style Pro HJA-750C, Omron Health Care Co., Ltd., Kyoto, Japan)	WHO Guidelines on Physical Activity, Sedentary Behaviour and Sleep for Children Under 5 Years of Age	Demographic correlates (including gender) were collected	Gender	Boys performed more MVPA than girls	Gender matters in meeting PA guideline
Larouche et al. [76]	Nº children: 594 Schools: 16 sites Country: Canada (Mean age 4.7 yrs)	Children activity was recorded during all day	7 consecutive days during 24 h	Accelerometer (Actical, Respironics, Murrysville, USA)	Canadian physical activity guidelines for the early years (aged 0–4 years) (3–4 year olds) New Canadian physical activity	Outdoor time was assessed by parent report	Outdoor time	Among 5–6 year olds, each additional hour spent outdoors was associated with an additional 10 minutes of MVPA and an	Outdoor time has a large effect on PA among 5–6 year olds

					guidelines (5–6 year olds)			increased likelihood of meeting the PA guidelins	
Kracht et al. [78]	Nº children: 107<br>Schools: 10 preschools<br>Country: USA<br>(Mean age 3.5 yrs)	Children activity was recorded during all day	7 consecutive days during 24 h (not considering water activities)	Accelerometer (GT3X+, ActiGraphCorp, Pensacola, USA)	Canadian 24-Hour Movement Guidelines for the Early Years (0-4 years)	Demographic correlates (including age, gender, race and poverty level) were collected<br>Body weight and height	Age, gender, race and poverty level<br>Weight status	There were no correlations between age, gender, race, poverty level and weight status and meeting PA guideline	Age, gender, race, poverty level and weight status do not matter in meeting PA guideline
Engberg et al. [79]	Nº children: 647<br>Schools: 8 preschools<br>Country: Finland<br>(Mean age 4.7 yrs)	Children activity was recorded during all day	7 consecutive days during 24	Accelerometer (ActiGraph wGT3X-BT, ActiGraphCorp, Pensacola, USA)	WHO Guidelines on physical activity, sedentary behaviour and sleep for children under 5 years of age	Subjective Happiness Scale (parents)	Parental happiness	Parental happiness scores did not associate with children's meeting PA guidelines	Parents who are happier have children with multiple healthy energy balance-related behaviors (including meeting PA guidelines)
Vale et al. [80]	Nº children: 509<br>Schools: preschool<br>Country: Portugal<br>(Mean age 5.2 yrs)	Children activity was recorded during all day	7 consecutive days during 24 h (not considering sleep time)	Accelerometer (ActiGraph GT1M, ActiGraphCorp, Pensacola, USA)	National Association for Sport and Physical Education. Active Start: A Statement of Physical activity Guidelines for	Demographic correlates (including gender and parental education) were collected	Gender<br>Parental education	Boys performed more PA and MVPA than girls<br>Children with parents in the highest education level were less active	Gender and parental education matter in meeting PA guideline

					Children Birth to Five Years (PA) [95] (MVPA)			than children from low and middle education level in week PA	
Vale et al. [81]	Nº children: 607<br>Schools: preschool<br>Country: Portugal<br>(Mean age XX)	Children activity was recorded during all day	7 consecutive days during 24 h (not considering sleep time)	Accelerometer (ActiGraph GT1M, ActiGraphCorp, Pensacola, USA)	Australian National Physical Activity Recommendations for children 0–5 years (PA)<br>Canadian Physical Activity Guidelines for the Early Years (aged 0-4 years) (MVPA)	Demographic correlates (including gender) were collected	Gender	Boys performed more PA and MVPA than girls	Gender matters in meeting PA guideline
Vale et al. [82]	Nº children: 916<br>Schools: preschool<br>Country: Portugal<br>(Mean age 5 yrs)	Children activity was recorded during all day	7 consecutive days during 24 h (not considering sleep time)	Accelerometer (ActiGraph GT1M, ActiGraphCorp, Pensacola, USA)	Australian National Physical Activity Recommendations for children 0–5 years (PA)<br>Canadian Physical Activity Guidelines for the	Demographic correlates (including gender) were collected<br>Steps count	Gender	Boys performed more PA and MVPA than girls<br>A significant correlation was observed between minutes of total PA and steps per day	Gender matters in meeting PA guideline<br>Preschool-aged children who accumulate less than 9000 steps per day may be considered insufficiently active

					Early Years (aged 0-4 years) (MVPA)				
Adeta et al. [83]	Nº children: 381; Schools: 18 urban kindergartens, 11 rural villages; Country: Ethiopia; Mean age: 4.2±0.6 yrs	Children activity recorded all day (24h)	5 consecutive days (24h, excl. water activities)	Accelerometer (ActiGraph wGT3X-BT)	WHO 24-h movement guidelines (2019)	Parent questionnaires (socio-demographics); Accelerometer data	Child sex, place of residence (urban/rural), parent education	Rural vs. urban: Higher odds of meeting screen time, sleep, and combined guidelines. No significant associations: Child sex or parent education.	Rural children show higher adherence. Urban interventions should target screen time reduction and sleep promotion.
Mota et al.	Nº children: 144; Schools: 7 Early Childhood Education Centers; Country: Brazil; Age: 1-year-olds (12–23mo, n=60), 2-year-olds (24–35mo, n=84)	24-h movement behaviors (PA, screen time, sleep) during daily routines	7 consecutive days, 24h (excl. water activities)	Accelerometer (Actigraph wGT3X)	WHO 24-h movement guidelines (2019)	Parent-reported screen time/sleep; Accelerometer-derived PA; Direct anthropometry; Age/sex recorded	Child sex, age group (1-year vs. 2-year)	Overall compliance: 21%. PA: 98.6% compliance (no sex/age differences). Screen time: Higher compliance in girls and 2-year-olds. Sleep: >60% compliance (no sex/age differences).	Strategies should prioritize reducing screen time (especially for boys/1-year-olds) while maintaining PA levels. Low screen time compliance is the primary barrier to overall guideline adherence.

Cristian et al. [84]	Nº 1,918; 115 early ECEC services; Australia; Mean age 3.3 yrs	Daily activities (24h monitoring)	7 consecutive days (24h, excl. sleep/water)	ActiGraph GT3X+ accelerometer	Australian 24-Hour Movement Guidelines	Parent surveys, environmental audits, Geographic Information Systems (GIS), educator surveys	Home yard (size, equipment, vegetation); ECEC environment (tree canopy, play equipment); Dog ownership	Home yard features (+) associated with PA; ECEC tree canopy/equipment (+) associated with outdoor time/PA; Dog ownership linked to ↓ social-emotional problems	Home/ECEC environments critically enable PA; Policies should prioritize outdoor spaces, natural features, and dog-friendly activities
Yin et al. [85]	Nº children: 205 (117 boys, 88 girls); Schools: 5 kindergartens; Country: China; (Mean age 4.8 ± 0.51 years)	Children activity recorded all day (24-hour movement behaviors)	7 consecutive days (valid ≥3 weekdays + 1 weekend day, ≥8h/day)	Accelerometer (Actigraph wGT3X-BT, waist-worn)	WHO 24-h movement guidelines (2019)	Demographic correlates (gender) collected; PA via accelerometer; screen time/sleep via parent questionnaires	Gender	Boys had higher MVPA than girls; only 26.8% met PA guidelines	Gender significantly influences MVPA adherence; interventions should target PA promotion and screen time reduction in Chinese preschoolers.
Palmer et al. [86]	Nº children: 240; Schools: 3 Head Start centers; Country: USA; Mean age: 3.9 years	PA during Head Start hours (school day: 8:30 AM–3:20/3:30 PM)	7 consecutive days (valid ≥2 school days, 410–420 min/day)	Accelerometer (Actigraph GT3X+ wrist-worn)	U.S. Department of Health and Human Services (USDHHS) 2018 (15 min PA/hour, 60 min MVPA,	Parent-reported sex/race; Head Start Body Start Play Space Assessment (PSA) playground assessments by researchers	Playground quality (PSA score), child sex	PSA scores not associated with PA outcomes. Boys had higher MVPA/Vigorous PA and greater odds of meeting	Playground quality (PSA) did not predict PA adherence, but sex disparities exist. Boys consistently more active. Girls' PA may decrease in higher-

					180 min total PA during Head Start)			180-min PA guideline. Trend: Higher PSA linked to lower odds of girls meeting PA guidelines.	quality play-grounds (e.g., shaded/sensory features). Future research should examine gender-specific playground design.
Sigmundová et al.	Nº children: 381 (190 girls, 191 boys); Schools: Kindergartens/primary schools; Country: Czechia (Mean age 6.5 yrs)	24-hour movement behavior (sleep, sedentary, physical activity) during daily routine	7 consecutive days, 24h	Accelerometer (ActiGraph wGT3X-BT for children, GT9X Link for parents)	WHO 24-h movement guidelines (age-specific: 3–4 yrs and ≥5 yrs)	Parent-reported anthropometrics; Family Socio-Economic Status (SES) questionnaire; Parent-reported screen time; Accelerometer-derived parental PA	Child: gender, age, excessive body weight; Parent: age, education, SES, excessive body weight, adherence to ≥2 guidelines	26.3% met all three guidelines; 45.4% met ≥2 guidelines. Maternal overweight/obesity reduced odds. Maternal university education and adherence to ≥2 guidelines increased odds. Paternal university education increased odds for all three guidelines.	Parents (especially mothers) are key gatekeepers for children's adherence. Maternal normal weight, higher education, and guideline adherence positively influence children. Public health efforts should educate parents across socioeconomic backgrounds.

Kolehmainen et al. [87]	Nº children: 282, Country: England, Age: 12-36 months (mean 21)	Physical activity during waking hours, except water-based activities	3-7 days	Accelerometer (ActiGraph GT3X+)	WHO guidelines (180 min/day)	Sociobehavioural context (parent actions), Environmental factors (time to safe outdoors, main carer work hours, maximum daily temperature)	Child factors (age, sex, mobility, social-cognitive capacity, Index of Multiple Deprivation decile, recruitment pathway)	Mobility capacity was the strongest predictor for both total and very active time; age was significant for very active time; 91% met WHO guidelines	Young children across developmental states can achieve recommended physical activity levels; inclusive, ambitious expectations are needed for all children, including those with developmental challenges
Yu et al. [88]	Nº children: 81 (48 boys, 33 girls); Schools: 3 kindergartens; Country: Hong Kong; (Mean age ~5 yrs)	Children activity recorded all day	7 consecutive days (valid ≥3 weekdays + 1 weekend day, ≥8h/day)	Accelerometer (Actigraph GT3X-BT wrist-worn)	WHO 24-h movement guidelines (2019)	Demographic correlates (gender); Screen time via parent report (Netherland Physical Activity Questionnaire [NPAQ]); Sleep via Children's Sleep Habit Questionnaire (CSHQ, parent report)	Gender	Boys had higher MVPA than girls. Only 32.1% met MVPA guideline	Gender significantly influences MVPA adherence; Low MVPA compliance highlights need for interventions targeting PA promotion and screen time reduction in Hong Kong preschoolers.
Ré et al. [89]	Nº children: 367 (194 girls, 173 boys); Schools: 4 preschools; Country: Brazil;	Children activity recorded all day	7 consecutive days, 24h (excl. sleep)	Accelerometer (ActiGraph GT3X+)	WHO 24-h movement guidelines	Parent questionnaires (maternal education, leisure preferences);	CRF, MC, maternal education, leisure activi-	CRF associated with meeting PA guidelines in both genders.	Gender and sociocultural factors (e.g., ball games) significantly influence PA. Policies

	Mean age: 4.8±0.6 (girls), 4.9±0.6 (boys) yrs					predictive power of cardiorespiratory fitness (CRF) shuttle run (CRF); TGMD-2 (MC); anthropometrics	ties (sedentary/active without balls/active with balls)	Active leisure with balls strongly predicted compliance. Boys of low-educated mothers had higher odds of compliance.	should promote gender-equal access to culturally relevant activities (e.g., soccer in Brazil), especially for girls.
Mwase-Vuma et al. [90]	Nº children: 797; Schools: 17 countries (5 lower middle income level [L-MIC], 5 upper income level [U-MIC], 7 high income m level; Country: Multinational; Mean age: 4.0 yrs	Children activity recorded all day	3–5 consecutive days, 24h (valid ≥1 day)	Accelerometer (activPAL)	WHO total physical activity (TPA) guideline (≥11,500 steps/day = 180 min/day)	Parent questionnaire (sociodemographics)	Sex, age, residential area, country income level, parent education	Girls and 4-year-olds less likely; rural residents and L-MIC children more likely	Surveillance/interventions should prioritize girls, older preschoolers, and urban/U-MIC contexts to improve global TPA adherence.
Cristian et al. [92]	Nº 1,918; 115 ECEC services; Australia; Mean age 3.3 yrs	Daily activities (24h monitoring)	7 consecutive days (24h, excl. sleep/water)	ActiGraph GT3X+	Australian 24-Hour Movement Guidelines	Parent surveys, environmental audits, GIS, educator surveys	Home yard (size, equipment, vegetation); ECEC environment (tree canopy, play equipment);	Home yard features (+) associated with PA; ECEC tree canopy/equipment (+) associated with outdoor time/PA; Dog ownership	Home/ECEC environments critically enable PA; Policies should prioritize outdoor spaces, natural features, and dog-friendly activities

							Dog ownership	linked to ↓ social-emotional problems	
Nusurupia et al. [94]	Nº children: 429; Schools: Preschools across 10 middle-income countries (LMIC); Country: Bangladesh, Zimbabwe, Vietnam, Sri Lanka, Indonesia, Papua New Guinea, Brazil, China, Malaysia, South Africa; Mean age: 4.3±0.4 yrs	24-h movement behaviors (PA, sedentary, sleep)	≥2 valid days (24-h wear; ≥6h waking hours)	Accelerometer (activPAL)	WHO 24-h movement guidelines (2019)	Parent questionnaire (screen time, restrained time); activPAL (PA, sleep); Country-specific urban/rural classification	Residential area (urban/rural), sex, age, parental education (SES)	Urban vs. rural differences: No significant difference in meeting PA guidelines. Rural children had higher TPA and steps. SES (parental education) significantly differed.	Urban-rural setting was not a significant correlate for meeting PA guidelines. Both settings showed low adherence. Public health strategies should target both settings in LMICs, especially given low overall compliance.

Table S3. The correlations between meeting physical activity guidelines and their effects.

**Ref.**	**Sample**	**What Are Children Doing?**	**Duration of Data Recording**	**Technology (Type and Model)**	**Reference Guideline**	**How Were the Other Variables Extracted?**	**Correlation Variables**	**Results**	**Conclusions and Practical Applications**
Christian et al. [47]	Nº children: 1363 Schools: 104 preschools Country: Australia (Mean age 3.3)	Children activity recorded all day	7 consecutive days, 24h (excl. sleep)	Accelerometer (ActiGraph GT3X+)	Australian 24-Hour	Strengths and Difficulties Questionnaire (SDQ)	Socioemotional (SDQ)	Lower difficulties in boys, no correlation in girls	Benefits for boys' emotional health

Nicolai Ré et al. [49]	Nº children: 1017 Schools: 3 preschools, elementary, middle Country: Brazil (Mean age 7.21)	Children activity recorded all day	7 consecutive days, 24h (excl. sleep/water)	Accelerometer (ActiGraph GT3X+)	WHO 24-h	TGMD-2 and Körperkoordinationstest für Kinder (KTK)	Motor competence (KTK)	No association with MC for most, except middle school boys	Focus on girls' MC decline
McNeill et al. [50]	Nº children: 247/185 Schools: preschool Country: Australia (Mean age 4.3)	Children activity recorded all day	7 consecutive days, 24h (excl. water)	Accelerometer (ActiGraph GT3X+)	Australian 24-Hour	Early Years Toolbox	Executive functions	Better phonological memory, shifting scores	Beneficial for cognitive health
Kracht et al. **[51]**	Nº children: 169 (cross-sectional)/107 (longitudinal) Schools: preschool Country: USA (Mean age 3.4 yrs)	Children activity was recorded during all day	7 consecutive days during 24 h (not considering water activities)	Accelerometer (ActiGraph GT3X+, ActiGraphCorp, Pensacola, USA)	WHO guidelines on physical activity, sedentary behaviour and sleep for children under 5 years of age	Test of Gross Motor Development-Third Edition (TGMD-3)	Raw locomotor, ball skills and total TGMD-3 scores	MVPA was positively associated with locomotor and total TGMD-3 scores	Meeting PA guideline resulted relevant in achieving gross motor ability scores
Meredith-Jones et al. **[52]**	Nº children: 547 Schools: N/A Country: New Zealand (Mean age 1, 2 and 5 yrs)	Children activity was recorded during all day	5-7 consecutive days during 24 h	Accelerometer (Actical, Respironics, Murrysville, USA)	Canadian 24-h movement guidelines for the early years (0–4 years)	Lunar Prodigy scanner (GE Medical Systems Lunar, Madison, USA)	Body composition at 5 yrs	Adherence to meeting guidelines was not related to body composition at age 5	Body composition does not matter in meeting PA guideline

O'Neill et al. **[53]**	Nº children: 341<br>Schools: 3 preschools<br>Country: USA<br>(Mean age 4.6 yrs	Children activity was recorded during all day	5 consecutive weekdays during 24 h (not considering sleep time or water activities)	Accelerometer (ActiGraph GT1M and GT3X, ActiGraphCorp, Pensacola, USA)	Early Childhood Obesity Prevention Policies	In-school and out-school time	In-school and out-school time	Children who met the guideline in-school remained comparably active out-of-school<br>Children who did not meet the guideline were more active out-of-school than in-school	Preschool and classroom factors may reduce some children's PA in-school
Guan et al. **[54]**	Nº children: 254<br>Schools: 3 preschools<br>Country: China<br>(Mean age 5.1 yrs)	Children activity was recorded during all day	3 consecutive weekdays during 24 h (not considering water activities)	Accelerometer (ActiGraph GT9X, ActiGraphCorp, Pensacola, USA)	WHO. Guidelines on physical activity, sedentary behaviour and sleep for children under 5 years of age	Body weight and height	Body mass index	Not meeting the physical activity guideline was not associated with overweight or obesity	There are not any associations between adiposity and the PA guideline
Leppänen et al. **[55]**	Nº children: 778<br>Schools: 66 preschools<br>Country: Finland<br>(Mean age 4.7 yrs)	Children activity was recorded during all day	7 consecutive days during 24 h (not considering water activities)	Accelerometer (ActiGraph wGT3X-BT, ActiGraphCorp, Pensacola, USA)	Australian 24-Hour Movement Guidelines for the early years (Birth to 5 years)	Body weight and height and waist circumference	BMI and waist circumference (WC)	Meeting guideline for PA was associated with lower WC<br>Meeting guideline for was associated	Meeting recommendation for PA may have an important role in supporting a healthy weight

								with lower BMI	status in young children
Santos et al. **[57]**	Nº children: 202<br>Schools: preschool<br>Country: Australia<br>(Mean age 1.6 yrs)	Children activity was recorded during all day	7 consecutive days during 24 h	Accelerometer (ActiGraph GT3X, ActiGraphCorp, Pensacola, USA)	Australian 24-Hour Movement Guidelines for the Early Years (Birth to 5 years)	Body weight and height	BMI	BMI was not associated with the accomplishment of the 24-h Movement Guideline	Further studies with more robust designs and larger samples are necessary to confirm or rule out the lack of association between meeting guideline and BMI
Cliff et al. **[58]**	Nº children: 248<br>Schools: preschool<br>Country: Australia<br>(Mean age 4.2 yrs)	Children activity was recorded during all day	7 consecutive days during 24 h	Accelerometer (ActiGraph GT3X, ActiGraphCorp, Pensacola, USA)	Australian 24-Hour Movement Guidelines for the Early Years (Birth to 5 years)	Test of Emotional Comprehension (TEC) and Theory of Mind (ToM)	Emotional understanding and capacity to understand other people by ascribing mental states to them	Children meeting the PA guideline performed better on TEC and ToM, than those who did not	Supporting preschool children to meet guideline may be beneficial for their social-cognitive development
Draper et al. **[59]**	Nº children: 88<br>Schools: 7 preschools<br>Country: South Africa<br>(Mean age 4.5 yrs)	Children activity was recorded during all day	3 consecutive days during 24 h (not considering sleep time or water activities)	Accelerometer (ActiGraph GT3X, ActiGraphCorp, Pensacola, USA)	WHO guideline for physical activity for children under five years of age	Body weight and height<br>Ages and Stages Questionnaire-3	BMI<br>Gross and fine motor skills	BMI was positively associated with PA<br>Gross motor skills were positively associated with both PA and MVPA	This study contributes to the growing literature on 24-hour movement behaviours in preschool children and highlights that these

								Fine motor skills were negatively associated with PA	behaviours require attention in this age group
Hall et al. **[61]**	Nº children: 166<br>Schools: preschool<br>Country: UK<br>(Mean age 4.28 yrs)	Children activity was recorded during all day	4 consecutive days during 24 h (not considering water activities)	Accelerometer (GENEActiv Activeinsights, Kimbolton, UK)	WHO Global Recommendations on Physical Activity for Health	TGMD-2	MC, Locomotor MC (LC) and object-control MC (OC)	Associations were found between PA and MC (PA and overall MC, PA and OC, MVPA and overall MC, and MVPA and OC)	Good motor competence is an important correlate of children meeting PA guideline
Breau et al. **[62]**	Nº children: 193<br>Schools: 61 preschools<br>Country: Germany<br>(Mean age 4.2 yrs)	Children activity was recorded during all day	7 consecutive days during 24 h (not considering sleep time or water activities)	Accelerometer (GENEActiv Activeinsights, Kimbolton, UK)	WHO guidelines on physical activity, sedentary behaviour and sleep for children under 5 years of age	Shuttle run, standing long jump, lateral jumping, one leg stand and sit and reach tests were administered	Shuttle run, standing long jump, lateral jumping, one leg stand and sit and reach tests performance	Meeting WHO guidelines were not significantly associated with performance on any of the motor ability tests	This study did not find any statistically significant associations between meeting PA guideline and motor abilities
Feng et al. **[63]**	Nº children: 251<br>Schools: 8 preschools<br>Country: China	Children activity was recorded during all day	7 consecutive days during 24 h	Accelerometer (activPAL3 micro or activPAL3activPAL3C vt,	WHO guidelines on physical activity, sedentary behavior, and	Body weight and height	BMI and odds ratio for being of normal weight	There was no significant association between meeting the PA guideline and body	Meeting the 24 24-hour movement guideline was not associated with a favourable

	(Mean age 5.1 yrs)			PAL Technologies, Glasgow, UK)	sleep for children less than 5 years of age			mass index or the odds ratio for being of normal weight	weight status among preschoolers
Armstrong et al. **[65]**	Nº children: 101<br>Schools: prechool<br>Country: UK<br>(Mean age 1.7 yrs)	Children activity was recorded during all day	3-7 consecutive days during 24 h	Accelerometer (Actical, Philips Respironics, Murrysville, USA)	Canadian 24-hour movement guidelines for the early years	Body weight and height<br>Demographic correlates (including poverty ratio) were collected	BMI z-score<br>Poverty ration	BMI z-score was not associated with meeting guideline<br>Poverty was associated with less than 60 minutes of MPVA	Objective measures of PA are needed to examine disparities among children from varying economic backgrounds
Berglind et al. **[66]**	Nº children: 830<br>Schools: preschool<br>Country: Sweden<br>(Mean age 4.3 yrs)	Children activity was recorded during all day	7 consecutive days during 24 h (not considering sleep time)	Accelerometer (ActiGraph GT3X+, ActiGraphCorp, Pensacola, USA)	Canadian 24-Hour Movement Guidelines for Children and Youth	Body weight and height	BMI and BMI z-score	Adherence to PA recommendation at age 4 years was not associated with being overweight or obese nor with BMI and BMI z-score at age 4 or 5 years	More prospective data are needed before effects on weight status from meeting the 24-h movement guidelines are elucidated
Carson et al. **[67]**	Nº children: 539<br>Schools: preschool	Children activity was recorded during all day	7 consecutive days during 24 h	Accelerometer (ActiGraph GT3X-BT,	Canadian 24-Hour Movement	Child Behavior Checklist	Externalizing, internalizing and total problem scores	Meeting recommendation was associated with lower	Meeting recommendation within the 24-

	Country: Canada (Mean age 3 yrs)			ActiGraphCorp, Pensacola, USA	ment Guidelines for the Early Years			scores for total, externalizing and internalizing problems	hour Movement Guidelines was associated with fewer behavioral and emotional problems at 3-years
Chaput et al. **[68]**	Nº children: 803 Schools: preschool Country: Canada (Mean age 3.5 yrs)	Children activity was recorded during all day	7 consecutive days during 24 h (not considering sleep time)	Accelerometer (Actical, Philips Respironics, Murrysville, USA)	Canadian 24-Hour Movement Guidelines for the Early Years (0-4 years)	Body weight and height	BMI z-score	No association was found between meeting PA recommendation and adiposity	Future work should examine the associations of guideline adherence with health indicators other than adiposity
Lee et al. **[71]**	Nº children: 230 Schools: 22 preschools Country: Malaysia (Mean age 5.5 yrs)	Children activity was recorded during all day	7 consecutive days during 24 h (not considering sleep time or water activities)	Accelerometer (Actical, Respironics, Murrysville, USA)	WHO Guidelines on Physical Activity, Sedentary Behaviour and Sleep for Children Under 5 Years of Age (children aged 4 yrs) WHO guidelines on physical activity	Body weight and height	Weight status	No association was found between meeting PA recommendation and weight status	Future studies looking at the association of movement behaviors with other obesity indicators, including metabolic syndrome and adiposity, should be considered

					and sedentary behaviour for children and adolescents aged 5–17 years (children aged 5-6 yrs)				
Li et al. **[73]**	Nº children: 322<br>Schools: 5 preschools<br>Country:China<br>(Mean age 4.7 [boys] and 4.6 [girls] yrs)	Children activity was recorded during all day	7 consecutive days during 24 h (not considering sleep time or water activities)	Accelerometer (ActiGraph wGT3X-BT, ActiGraphCorp, Pensacola, USA)	WHO Guidelines on physical activity, sedentary behaviour and sleep for children under 5 years of age	TGMD-3	Raw locomotor, ball skills and total TGMD-3 scores	There were positive associations between meeting the PA guidelines and both locomotor and ball skills	PA levels, especially MVPA, are important for the promotion of fundamental movement skills
Kim et al. **[74]**	Nº children: 421<br>Schools: 6 preschools<br>Country: Japan<br>(Mean age 4.6 yrs)	Children activity was recorded during all day	7 consecutive days during 24 h (not considering sleep time or water activities)	Accelerometer (Active Style Pro HJA-750C, Omron Health Care Co., Ltd., Kyoto, Japan)	WHO Guidelines on Physical Activity, Sedentary Behaviour and Sleep for Children Under 5 Years of Age	Body weight and height	Weight status	There was no association between meeting the PA guideline and weight status	Screen time and sleep have to take into account, as well
Kim et al. **[75]**	Nº children: 103<br>Schools: 4 preschools<br>Country: Vietnam	Children activity was recorded during all day	3 consecutive days during 24 h (not considering water activities)	Accelerometer (GT3X+, ActiGraphCorp, Pensacola, USA)	WHO Guidelines on Physical Activity, Sedentary Behaviour and	Body weight and height<br>Ages and Stages Questionnaire (3rd edition) for	Weight status<br>Gross and fine motor skills	There was no association between guideline compliance and adi-	Although relationships are not yet found between guideline compli-

	(Mean age 4.1 yrs)				Sleep for Children Under 5 Years of Age	children aged 48 mo		posity and motor development	ance and adiposity and motor development, health promotion programs should focus on encouraging children to be more active, especially to spend more time on light-intensity PA
Lee et al. **[77]**	Nº children: 151<br>Schools: 4 centres<br>Country: Canada<br>(Mean age 1.6 yrs)	Children activity was recorded during all day	7 consecutive days during 24 h (not considering sleep time or water activities)	Accelerometer (ActiGraph wGT3X-BT, ActiGraphCorp, Pensacola, USA)	Canadian 24-Hour Movement Guidelines for the Early Years (0–4 years): An Integration of Physical Activity, Sedentary Behaviour, and Sleep	Body weight and height	BMIz	No associations were observed between meeting PA recommendation within the guidelines and BMIz	Future research should examine the associations between meeting the new guideline and other health indicators
Taylor et al.	Nº children: 528<br>Schools: n/a<br>Country: New Zealand	Children activity was recorded during all day	7 consecutive days during 24	Accelerometer (Actical, Respironics, Murrysville, USA)	Canadian 24-hour movement guidelines for the early years (0–4 years)	Anxiety, depression and resilience subscales of the Behavioral Assessment System for	Anxiety, depression and resilience at 5 yrs of age	Children who met PA guidelines at 1 year of age had lower anxiety and depression	Future work should consider a compositional approach to 24-h time use and

	(Mean age 1, 2, 3.5, and 5 yrs)					Children (BASC-2) 2–5 year old scale at 5 yrs of age		scores at 5 yrs of age than those who did not	how it may influence mental wellbeing
Vale et al. **[81]**	Nº children: 607<br>Schools: preschool<br>Country: Portugal<br>(Mean age XX)	Children activity was recorded during all day	7 consecutive days during 24 h (not considering sleep time)	Accelerometer (ActiGraph GT1M, ActiGraphCorp, Pensacola, USA)	Australian National Physical Activity Recommendations for children 0–5 years (PA)<br>Canadian Physical Activity Guidelines for the Early Years (aged 0-4 years) (MVPA)	Body weight and height	Obesity status	Not meeting the MVPA guideline was associated with obesity status in girls, but not boys	There is an association with low levels of MVPA and higher obesity status among preschool girls
Cristian et al. **[84]**	Nº 1,918; 115 ECEC services; Australia; Mean age 3.3 yrs	Daily activities (24h monitoring)	7 consecutive days (24h, excl. sleep/water)	ActiGraph GT3X+ accelerometer	Australian 24-Hour Movement Guidelines	Strengths and Difficulties Questionnaire (SDQ)	Social-emotional development (SDQ)	Meeting all guidelines (+) associated with boys' social-emotional development (no association in girls)	Meeting guidelines benefits boys' socioemotional health; Gender-specific interventions needed

Yin et al. **[85]**	Nº children: 205 (117 boys, 88 girls); Schools: 5 kindergartens; Country: China; (Mean age 4.8 ± 0.51 years)	Children activity recorded all day (24-hour movement behaviors)	7 consecutive days (valid ≥3 weekdays + 1 weekend day, ≥8h/day)	Accelerometer (Actigraph wGT3X-BT, waist-worn)	WHO 24-h movement guidelines (2019)	Mental health (SDQ: prosocial, externalizing, internalizing) via parent report; PA via accelerometer; covariates (age, gender, BMI, SES)	Prosocial behaviour, externalizing problems	Meeting PA guideline → ↑ prosocial behaviour; Meeting all guidelines → ↑ prosocial behaviour; Meeting ST + sleep guidelines → ↓ externalizing problems	PA enhances prosocial behaviour; combined adherence to guidelines improves mental health; interventions should prioritize holistic 24-h movement behaviours.
Palmer et al. **[86]**	Nº children: 240; Schools: 3 Head Start centers; Country: USA; Mean age: 3.9 years	PA during Head Start hours	7 consecutive days (valid ≥2 school days)	Accelerometer (Actigraph GT3X+ wrist-worn)	USDHHS 2018 (180 min total PA during Head Start)	Parent-reported sex; PSA assessments	Playground quality (PSA score), child sex	Meeting 180-min guideline: No association with PSA scores. Boys had higher odds of meeting guidelines. Girls: Higher PSA scores trended toward lower odds.	Playground quality does not affect PA guideline adherence. Boys more likely to meet guidelines regardless of environment. Girls' PA may be inhibited by "high-quality" features (e.g., sheltered sandboxes).

									Policies should prioritize gender-inclusive playground designs.
Kolehmainen et al. **[87]**	Nº children: 282 Schools: N/A Country: England, Age: 12-36 months (mean 21)	Physical activity during waking hours, except water-based activities	3-7 days	Accelerometer (ActiGraph GT3X+)	WHO guidelines (180 min/day)	N/A	Physical activity levels and health-related quality of life (HRQoL [Pediatric Quality of Life Inventory])	No evidence that physical activity explains HRQoL in multivariable analysis	Further research is needed to understand the relationship between physical activity and HRQoL in young children
Ré et al. **[89]**	Nº children: 367 (194 girls, 173 boys); Schools: 4 preschools; Country: Brazil; Mean age: 4.8±0.6 (girls), 4.9±0.6 (boys) yrs	Children activity recorded all day	7 consecutive days, 24h (excl. sleep)	Accelerometer (ActiGraph GT3X+)	WHO 24-h movement guidelines	Parent questionnaires; CRF/MC tests; anthropometrics	Meeting PA guidelines × CRF, MC, leisure activities	Meeting guidelines linked to higher CRF in both genders. Active leisure with balls increased compliance odds. No MC differences between leisure groups.	CRF and sociocultural factors (leisure preferences) are stronger predictors of PA compliance than MC. Interventions should target gender-specific barriers and

									promote active leisure (e.g., ball games).
Byambaa et al. **[91]**	Nº children: 101 (58 boys, 43 girls) Schools: 5 preschools Country: Mongolia (Mean age 3.97)	Recorded 24 h/day (waking time)	5 consecutive days (24 h wear per day)	Waist-worn ActiGraph accelerometer (GT3X+/GT3X-BT)	WHO 24-h movement guidelines for <5 y (PA, sedentary behavior, sleep)	Parent-reported sleep/screen time; NIH Early Years Toolbox (motor skills); mass/height	Gender (sex); Urban vs. rural location; Motor skill scores	77% of boys vs 39% of girls met the WHO PA guideline; no significant urban–rural difference in adherence. Children meeting both PA and sleep guidelines had higher gross and fine motor skill scores	Boys were more likely than girls to meet PA recommendations; intervention may need to emphasize PA for girls. Meeting guidelines was associated with better motor development (gross/fine skills), highlighting the benefit of adhering to PA/sleep guidelines.
Christian et al. **[92]**	Nº 1,918; 115 ECEC services; Australia; Mean age 3.3 yrs	Daily activities (24h monitoring)	7 consecutive days (24h, excl. sleep/water)	ActiGraph GT3X+	Australian 24-Hour Movement Guidelines	Strengths and Difficulties Questionnaire (SDQ)	Social-emotional development (SDQ)	Meeting all guidelines (+) associated with boys' social-emotional development	Meeting guidelines benefits boys' socio-emotional health; Gender-

								(no association in girls)	specific interventions needed
Li et al. **[93]**	Nº 109 children; 3 kindergartens; China; Mean age: 4.3±0.6 yrs	Children activity recorded all day	7 consecutive days, 24h (excl. sleep/water)	Accelerometer (ActiGraph wGT3X-BT); BMD via ultrasound (Sunlight Omnisense 7000P)	WHO 24-h movement guidelines	BMD z-scores; covariates adjusted in regression	Bone Mineral Density (BMD)	Cross-sectionally: Meeting screen time+sleep duration reduced insufficient BMD risk. Longitudinally: Meeting PA or all guidelines reduced risk.	Adherence to 24-h guidelines promotes bone health. Prioritize limiting screen time, ensuring sleep, and gradually increasing MVPA.
